# Designing Short Peptides to Block the Interaction of SARS-CoV-2 and Human ACE2 for COVID-19 Therapeutics

**DOI:** 10.3389/fphar.2021.731828

**Published:** 2021-08-27

**Authors:** Abdul Basit, Asad Mustafa Karim, Muhammad Asif, Tanveer Ali, Jung Hun Lee, Jeong Ho Jeon, Shafiq ur Rehman, Sang Hee Lee

**Affiliations:** ^1^ Institute of Microbiology and Molecular Genetics, University of the Punjab, Lahore, Pakistan; ^2^ Department of Bioscience and Biotechnology, The University of Suwon, Hwaseong, South Korea; ^3^ Department of Host Defense, Graduate School of Medicine, University of the Ryukyus, Nishihara, Japan; ^4^ National Leading Research Laboratory, Department of Biological Sciences, Myongji University, Yongin, South Korea

**Keywords:** COVID-19, SARS-CoV-2, RBD, designed peptide, s glycoprotein

## Abstract

To date, the current COVID-19 pandemic caused by SARS-CoV-2 has infected 99.2 million while killed 2.2 million people throughout the world and is still spreading widely. The unavailability of potential therapeutics against this virus urges to search and develop new drugs. SARS-CoV-2 enters human cells by interacting with human angiotensin-converting enzyme 2 (ACE2) receptor expressed on human cell surface through utilizing receptor-binding domain (RBD) of its spike glycoprotein. The RBD is highly conserved and is also a potential target for blocking its interaction with human cell surface receptor. We designed short peptides on the basis of our previously reported truncated ACE2 (tACE2) for increasing the binding affinity as well as the binding interaction network with RBD. These peptides can selectively bind to RBD with much higher affinities than the cell surface receptor. Thus, these can block all the binding residues required for binding to cell surface receptor. We used selected amino acid regions (21–40 and 65–75) of ACE2 as scaffold for the *de novo* peptide design. Our designed peptide Pep1 showed interactions with RBD covering almost all of its binding residues with significantly higher binding affinity (−13.2 kcal mol^−1^) than the cell surface receptor. The molecular dynamics (MD) simulation results showed that designed peptides form a stabilized complex with RBD. We suggest that blocking the RBD through *de novo* designed peptides can serve as a potential candidate for COVID-19 treatment after further clinical investigations.

## Introduction

Severe acute respiratory syndrome-coronavirus 2 (SARS-CoV-2) is the enveloped and positive-stranded RNA virus (Muralidharan et al., 2021). SARS-CoV-2 was emerged and started causing coronavirus disease 2019 (COVID-19). Hence, it is the utmost public health emergency at present with no treatment available so far, with an urgent need of potent drug against COVID-19 (Muralidharan et al., 2021). Currently, SARS-CoV-2 has affected the whole world and possibly it can re-emerge in the future with some virus beneficial mutations which might lead to more-worst outcome. Coronaviruses use spike (S) glycoprotein to attach and fuse with host cells, followed by entry into the cell. The interaction between the receptor-binding domain (RBD) of S protein and the human angiotensin-converting enzyme 2 (ACE2) happens while the S protein is in the pre-fusion conformation. The binding of the S protein in pre-fusion conformation with ACE2 triggers the cleavage of the S protein in two large domains: the N-terminal domain that remains attached to ACE2 and the C-terminal domain which folds in the so called post-fusion conformation (6-helix bundle fusion core) determining host-cell invasion (viral membrane fusion process) (Mercurio et al., 2021). A recent study has diagnosed SARS-CoV-2 in serum, urine and fecal samples with a low detection rate (Kim et al., 2020; Wang et al., 2020). Although it is challenging to determine whether the urinary tract, bladder or blood cells are also infected by SARS-CoV-2, virtual screening of RBD with cell surface receptor can raise the possibility of fecal/urine-respiratory infection.

Interestingly, the SARS-CoV-1 and -2 bind with cell surface receptor through RBD (a highly conserved region of S protein) (Singh et al., 2020), which suggests this domain a suitable target for drug designing (Lizbeth et al., 2020). The structural insights of SARS-CoV-2 and ACE2 interactions have been extensively studied (Lan et al., 2020; Yan et al., 2020). The RBD residues critical for interaction with ACE2 are located at position 417, 458, 493–498, and 500–502 (Chan et al., 2020; Lan et al., 2020; Yan et al., 2020; Basit et al., 2021). This suggests that almost similar binding residues of RBD are used to interact with cell surface receptor. The overall sequence of RBD is highly conserved with more than 99.9% homology with worldwide sequences of RBD reported (Basit et al., 2021). Structural elucidation has also confirmed the highly conserved nature of RBD (Lan et al., 2020). Blocking the binding residues of RBD can impede the SARS-CoV-2 to infect the human cells (Huang et al., 2020). The interactions between RBD and cell surface receptor have been extensively elucidated (Chan et al., 2020; Wan et al., 2020; Yan et al., 2020), which can be exploited to design peptide-based inhibitors targeting binding residues of RBD. Several studies have reported peptides for blocking the fusion of SARS-CoV-2 RBD with human cell surface receptor and for targeting the HR1 domain, which have shown successful inhibitory effects (Du et al., 2009; Xia et al., 2019; Han and Kral, 2020; Karoyan et al., 2021). Previous studies have shown that the residues of ACE2 at position 21–40 and 76 are optimal for binding with RBD (Huang et al., 2020; Basit et al., 2021). There are several other peptides reported for blocking RBD of SARS-CoV-2 and SARS-CoV-1 (Han et al., 2006; Chan et al., 2020). However, these peptides may not cover all the binding residues of RBD. Engineering the optimal regions of ACE2 and expanding their binding interaction network can significantly block the infection of SARS-CoV-2 into human cells. *De novo* protein design is a novel approach used to optimize the binding interface of protein-protein interactions by mutating the residues into favorable mutants which provide new binding interactions with increased binding affinity and preserved secondary structure (Chevalier et al., 2017). Recently, Huang et al. (2020), redesigned the previously reported two natural peptides from ACE2 through EvoDesign (Pearce et al., 2019) and produced a hybrid peptide with improved binding affinity for RBD and showed interactions with residues Y453, F456, Y473, A475, N487, and Y489 of RBD.

In the current study, we aimed to design peptides on the basis of our previously reported truncated ACE2 (tACE2) (Basit et al., 2021) by using EvoDesign, a *de novo* peptide design approach, to increase not only the binding affinity but also extend the binding interaction network with RBD. We have selected two regions of ACE2 (21–40 and 65–75) as a template for *de novo* peptide design (Wan et al., 2020). We designed two peptides, Pep1 and Pep2 for binding with RBD and determined their binding affinity and complex stability through protein-protein docking and molecular dynamics (MD) simulations. The present study will open a new path for designing therapeutic peptides against COVID-19.

## Materials and Methods

### Designing COVID-19 Therapeutic Peptides

The three-dimensional (3D) structure (protein data bank [PDB] ID: 6m17) of RBD of SARS-CoV-2 S glycoprotein was obtained from PDB database. Two peptides (Pep1 and Pep2) were deigned against the binding residues at position 417, 453, 458, 493–498, and 500–505 of RBD (Yan et al., 2020). The amino acid position 21–40 of tACE2 binds with the binding residues 493–498 and 501–505 of RBD (Basit et al., 2021), while 65–75 amino acid region of tACE2 interacts with binding residues 417, 453. and 458 of RBD (Lizbeth et al., 2020). Therefore, we selected these two fragments of ACE2 from amino acid position 21–40 and 65–75 as scaffold1 and scaffold2, respectively, for *de novo* peptide design to further enhance their binding affinity for RBD. The 3D structure of the scaffold peptides were produced through I-TASSER (Yang et al., 2015) and optimized for energy minimization through FoldX (Schymkowitz et al., 2005). The optimized scaffold structures were submitted as template to EvoDesign server (https://zhanglab.ccmb.med.umich.edu/EvoDesign/) using interface design. The template modeling-score (TM-score) >0.5 indicates that the designed peptide has similar fold to that of scaffold while the value < 0.2 correspond to those of randomly chosen unrelated proteins (Pierce et al., 2014). EvoDesign outputs the top 10 sequences selected from the largest clusters. The top ten designed sequences obtained for each peptide was sorted based on TM-score, sequence identity and lowest free energy. The sequence with the lowest free energy was considered as favorable design. However, we selected Pep1 and Pep2 from their corresponding top 10 sequences based on their Z-score and HADDOCK-score calculated by HADDOCK server (https://wenmr.science.uu.nl/haddock2.4/). The 3D models of the designed peptides were produced through I-TASSER (Yang et al., 2015). The selected designed peptides were analyzed for their fold similarity through template modeling alignment (TM-align) (Zhang and Skolnick, 2005).

### Docking of RBD With Designed Peptides

Protein-protein docking of the designed peptides with RBD was performed through HADDOCK, a flexible protein-protein docking tool (van Zundert et al., 2016). The structures of designed peptides were optimized before docking for amino acid side chain clashes and energy minimization by using FoldX (Schymkowitz et al., 2005). HADDOCK performs protein-protein docking by retrieving information from experimentally determined protein-protein complexes. The energy function used by HADDOCK consists on combination of interaction energies and HADDOCK-score, which is a combination of non-bonded intermolecular interactions (Vangone et al., 2017). All the generated docking poses were analyzed through PyMOL (Schrodinger, 2010). The best docked complex of RBD with designed peptides were selected on the basis of HADDOCK-score and were further analyzed for binding affinity ΔG (kcal mol^−1^) and complex stability by using an online protein binding energy prediction server (https://bianca.science.uu.nl/prodigy/), PRODIGY (Xue et al., 2016). Dissociation constant Kd (M) was determined as previously described (Basit et al., 2021). The peptides-RBD docked complexes with higher binding affinity were subjected to MD simulation to further confirm complex stability.

### Determination of RBD-Peptide Complex Stability Through MD Simulation

MD simulation of RBD in complex with designed peptides (Pep1 and Pep2) was performed through GROMACS 5.0.4 (Van Der Spoel et al., 2005; Abraham et al., 2015) using CHARM 27.0 force field (Huang and MacKerell, 2013). The protein complex was solvated in TIP3P cube box water model (volume: 596.38 nm^3^ and density: 994.63 g L^−1^) to provide an aqueous environment with a total 55,386 water molecules. The protein complex was centered in the box with a distance of at least 1.0 nm from the simulation box edge, while 1.0 nm distance between the atoms with non-bonded interactions was maintained. To neutralize the total charge of the system, one Cl^−^ ion was added to the box followed by energy minimization to remove conflict between the atoms (Ross et al., 2018). The system now containing 3141 protein atoms in addition to one Cl^−^ ion and 55,386 water molecules, was subjected to energy minimization using steepest descent method for 20,000 steps and then equilibrated through canonical ensemble (NVT: moles (N), volume (V) and temperature (T)) and isothermal-isobaric ensemble (NPT: moles (N), pressure (P) and temperature (T)) at constant temperature (300 K) and pressure (1 bar), respectively for 100 ps. Particle Mesh Ewald (PME) with grid spacing 0.16 nm were used for long-range electrostatics (Huang and MacKerell, 2013). MD simulation was then run for 100 ns at 300 K. Root mean square deviation (RMSD), root mean square fluctuation (RMSF)and radius of gyration (Rg) plots were produced through gnuplot (http://www.gnuplot.info/).

## Results and Discussion

### *De Novo* Design of Inhibitory Peptides Against RBD

RBD of spike glycoprotein mediates the entry of SARS-CoV-2 into the human respiratory cells by interacting with cell surface receptor ACE2 (Lan et al., 2020). Therefore, blocking the interaction residues of RBD might block its interaction with ACE2, hence making it unable to infect human cells. The RBD of SARS-CoV-2 and SARS-CoV-1 is highly conserved (Li et al., 2020) and mainly uses residues 417, 453, 458, 490, 493–495, 498, 501, and 502 for binding to ACE2 (Lan et al., 2020; Yan et al., 2020). Therefore, blocking the binding residues of RBD through inhibitory peptides can potentially block entry of SARS-CoV-2 into the human cells and can also be useful against future pandemic if caused by newly emerged coronaviruses due to the conserved nature of RBD (Lan et al., 2020). Thereby, targeting the RBD to block its interaction with ACE2 is ideal choice for SARS-CoV-2 drug discovery. At present, much research has been focused on non-invasive routes such as nasal, pulmonary, oral, ocular, and rectal for administering peptides (Ibraheem et al., 2014). Unfortunately, the widespread use of peptides as drugs is still faced by many obstacles such as low bioavailability, short half-life in the blood stream, *in vivo* instability, and numerous other problems. In order to overcome these hurdled and improve peptide drug efficacy, various strategies have been developed such as permeability enhancement, enzyme inhibition, and protection by encapsulation (Ibraheem et al., 2014).

Previously, we targeted these nine residues of RBD to be blocked through tACE2 (Basit et al., 2021). However, the current study involved re-designing the binding interface of tACE2 to produce shorter peptide with more binding affinity and covering all the binding residues of RBD (Fosgerau and Hoffmann, 2015). Short therapeutic peptides have gain interest because they have many advantages, such as low molecular weight, selectivity for a specific target, cells with minimal toxicity (Ellert-Miklaszewska et al., 2017). Furthermore, the use of chimeric peptides encompassing disease-targeting and cell-penetrating elements can increase specificity and efficacy of drug delivery together with reducing toxicity (Ellert-Miklaszewska et al., 2017).

The RBD binding residues 490, 493–495, 498, 501, 502 are clustered at one region (region1) while 417 and 458 are clustered at the other region (region2). Therefore, either two peptides can block these two regions or single peptide with extended binding network can hinder interaction between RBD and cell surface receptor.

**FIGURE 1:**
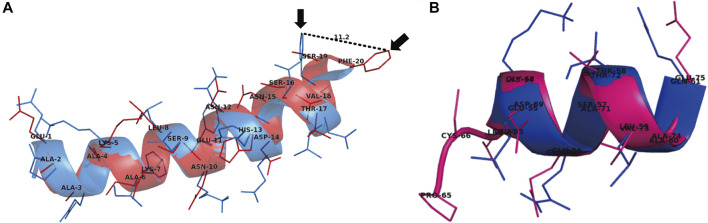
Superimposed models of *de novo* designed peptides showing comparison of their secondary structures to those of two scaffolds (selected amino acid regions [21–40 and 65–75] of ACE2) structure. **(A)** The Pep1 (red) superimposed on tACE2 showed almost similar secondary structure with C-α backbone RMSD 0.58 Å to the wild type tACE2. However, the changes (arrows) in positions of Pep1 residues’ R groups with respect to scaffold (blue) were observed, as shown the Phe20 side chain of ACE2 is moved 11.2 Å away from the Phe20 side chain of Pep1, which provide a favorable position for binding with Lys458 of RBD of SARS-CoV-2 spike glycoprotein. **(B)** The Pep2 (pink) showed notable different secondary structure composition from scaffold (dark blue) with C-α backbone RMSD 2.12 Å.

The residues of ACE2 at amino acid position 21–40 (scafold1) and 65–75 (scafold2) were re-designed and produced 10 *de novo* sequences for each scaffold. Two best sequences (Pep1 and Pep2) were selected from top-10 *de novo* sequences produced by EvoDesign from scaffold1 and scaffold2, respectively. The TM-score 0.61 of Pep1 (those of Pep3-10) indicate its similar fold to that of scaffold1, while Pep2 TM-score was 0.16 indicating its different fold than the scaffold2 structure. The Lower RMSD of Pep1 (0.58 Å) is in agreement with its TM-score, while Pep2 showed RMSD 2.12 Å, which indicate slight deviation of secondary structure from its scaffold (Figure 1). Similarly, the amino acid sequence of Pep1 showed 30% similarity while Pep2 showed 20% similarity with its corresponding native sequence (Table 1). The designed peptides with high similarity to their native sequence usually exhibit higher binding affinity towards its partner protein (Huang et al., 2020). We further investigated the binding pattern and affinity of the designed peptides for RBD through protein-protein docking.

**TABLE 1:** Summary of the *de novo* designed peptides produced by using ACE2 as scaffold.

Designed peptide	Sequence	TM-score^a^	Sequence identity (%)	RMSD^b^	Binding affinity (kcal mol^−1^)	Number of binding residues in RBD covered by the designed peptide
tACE2 fragment (scaffold1)	I_21_EEQAKTFLDKFNHEAEDLF_40_	—	—	—	−10.2	7
Pep1	EAAAKAKLSNENHDNSTVSF	0.61	30	0.58	−13.2	11
tACE2 fragment (scaffold2)	A_65_GDKWSAFLKE_75_	—	—	—	−7.6	3
Pep2	PCLGDQATVAE	0.16	20	2.12	−9.2	3
Pep3	EEAAKTTLANENSDNCFLSF	0.68	40	0.68	−12.8	10
Pep4	EQAAKATLANENSDNGFLSF	0.64	30	0.51	−11.2	9
Pep5	ESAAKAQLRQEDTENAAVMY	0.60	30	0.58	−11.8	8
Pep6	EAAAKSILSNENNDNSTASF	0.62	25	0.60	−10.92	7
Pep7	EENSCSFLAALFSEASCQSK	0.65	30	0.48	−11.8	8
Pep8	EFQQGCFISAADNCQSEISY	0.50	20	0.55	−11.5	8
Pep9	EKLTYSALQAEKTSSSPQSG	0.58	10	1.8	−10.8	6
Pep10	EHHAASKLMGIDQESAMIAL	0.61	20	0.78	−12.3	8

a Template modeling-score (TM-score) indicates the fold similarity between two structures (each peptide and ACE2). A TM-scores >0.5 correspond to almost similar fold while the value < 0.2 indicate randomly chosen unrelated proteins.

b Root mean square deviation (RMSD) calculated by TM-align shows the structural variations of two superimposed structures (each peptide and ACE2).

### Protein-Protein Docking

To test the binding properties, protein-protein docking of the designed peptides with RBD was performed through HADDOCK. The HADDOCK-scores (the more negative the better binding affinity) of Pep1 and Pep2 were −119 and −111, respectively, when docked with RBD. The HADDOCK-score of Pep1 is greater than that of the intact ACE2 (−111) docked with RBD (Basit et al., 2021). The docking RMSD of Pep1 and Pep2 in complex with RBD were 0.6 and 0.8, respectively, showing the high likelihood of the docked complexes with native one (Vangone et al., 2017).

**FIGURE 2:**
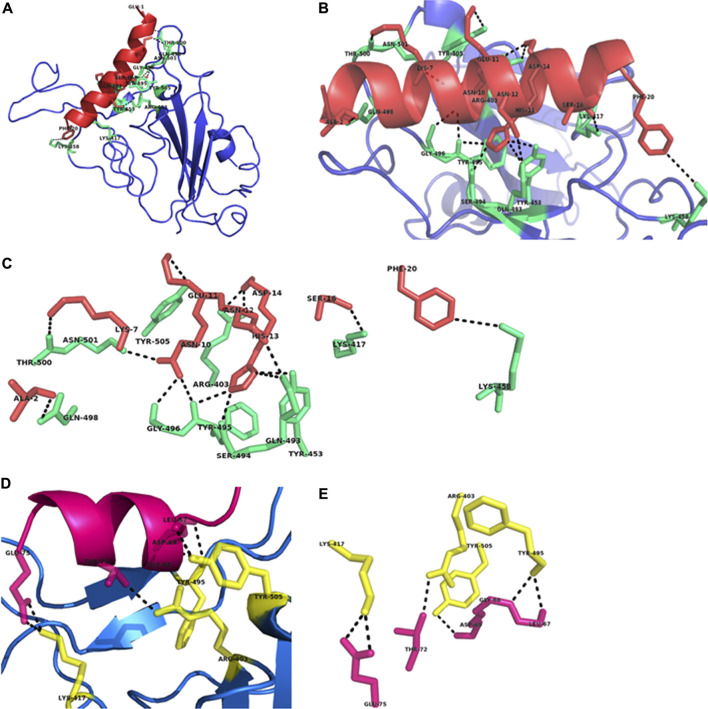
Structural analysis of the designed peptides (Pep1 and Pep2) in complex with RBD of SARS-CoV-2 spike glycoprotein. **(A)** Pep1-RBD complex shows the positioning of designed peptide Pep1 (red) in the binding interface of RBD (blue). **(B)** Pep1 comprises on a single helical structure (red) showing their interactions with the RBD binding residues shown in green. **(C)** The residues of Pep1 involved in binding interactions with RBD residues (green). **(D)** Pep2-RBD complex shows the positioning of Pep2 (pink) at the binding interface of RBD (blue). **(E)** The residues of Pep2 involved in binding interactions with RBD residues (yellow). All interactions are denoted by black lines.

Our docking results showed that nine residues Ala2, Lys7, Ans10-Asp14, Ser16, and Phe20 of Pep1 interact with Arg403, Lys417, Tyr453, Lys458, Gln493-Gly496, Gln498, Thr500, Asn501, and Tyr505 residues of RBD (Figures 2A–C), while Leu67-Asp69, Thr72 and Glu75 of Pep2 interact with Arg404, Lys417, Tyr495 and Tyr 505 of RBD (Figures 2D,E). Similarly, seven residues of wild type tACE2 scaffold (Glu23, Glu24, Lys31, His34, Glu35, Glu37, and Asp38) showed binding interactions with Seven residues (Tyr453, Lys458, Asn487, Tyr489, Gln498, Thr500 and Gly502) of RBD **(** Supplementary Figure S1 **).** These results confirm that Pep1 not only cover 11 the binding residues of RBD involved in interaction with human ACE2 (Table 1) but also other residues at position 403, 417, and 493–498, that may involve in interaction with human receptors, making this peptide ideal for further clinical investigation for its therapeutic potential.

Previous studies have shown that binding residues of RBD are located at two distinct position, region1 (490, 493–495, 498, 501, 502) and region2 (417 and 458) (Lan et al., 2020; Basit et al., 2021). Interestingly, our *de novo* designed peptide Pep1 showed binding with region1 as well as region2 residues (Figure 2C). The superimposition of docked Pep1 with its scaffold showed that redesigning moved the Phe20 into the favorable position for interaction with Lys458 of RBD, while mutation Ala16Ser results in interaction with Lys417 of RBD (Figures 1, 2C). Both of these residues are located at region2 and reported to be critical for interaction with RBD (Lan et al., 2020). The superimposition of designed peptides Pep3-10 with scaffold showed average RMSD 0.2 A˚, suggesting their almost similar C-α backbone with deviation in R group positioning **(** Supplementary Figure S2 **).** The *de novo* design approach created optimum mutation which increased binding network of the designed peptide Pep1, resulting in successful blocking of the RBD binding residues required for interaction with human cell surface receptor.

### Binding Affinity of Designed Peptides for ACE2

We further determined the binding affinity of the designed peptides for RBD and complex stability. The binding affinity showed by Pep1 for RBD was −13.2 kcal mol^−1^ at 36°C as optimum temperature which is greater than the binding affinity of wild type tACE2 (−10.7 kcal mol^−1^) (Basit et al., 2021) and scaffold tACE2 (−11.2 kcal mol^−1^). It seems that the favorable mutations and side chain rearrangement resulted in dramatic increase in binding affinity of Pep1 for RBD. The binding affinity of other designed peptides (Pep3-10) with RBD was found lower than Pep1 and almost higher than the scaffold (Table 1). We further determined the dissociation constant K_d_ values of peptide-receptor complexes. The Pep1-RBD complex showed K_d_ value 3.9 × 10^−10^ M, which is lower than the previously reported K_d_ values of inhibitory peptide (P8: 2.4 × 10^−9^ M) proposed for S protein of SARS-CoV-2 (Karoyan et al., 2021) and wild type tACE2-RBD complex (Basit et al., 2021). The smaller K_d_ value indicates high stability and strong binding affinity between protein-protein complex (Johnson et al., 2007). The lower K_d_ value of Pep1-RBD complex suggest that the designed peptide Pep1 are tightly bound to the corresponding region of RBD. Binding affinity of Pep2-RBD complex was found lower than the Pep1-RBD complex. This indicates that region 21–40 of tACE2 has important role in binding with RBD.

### MD Simulation Showed Stability of Designed Peptides-RBD Complex

**FIGURE 3:**
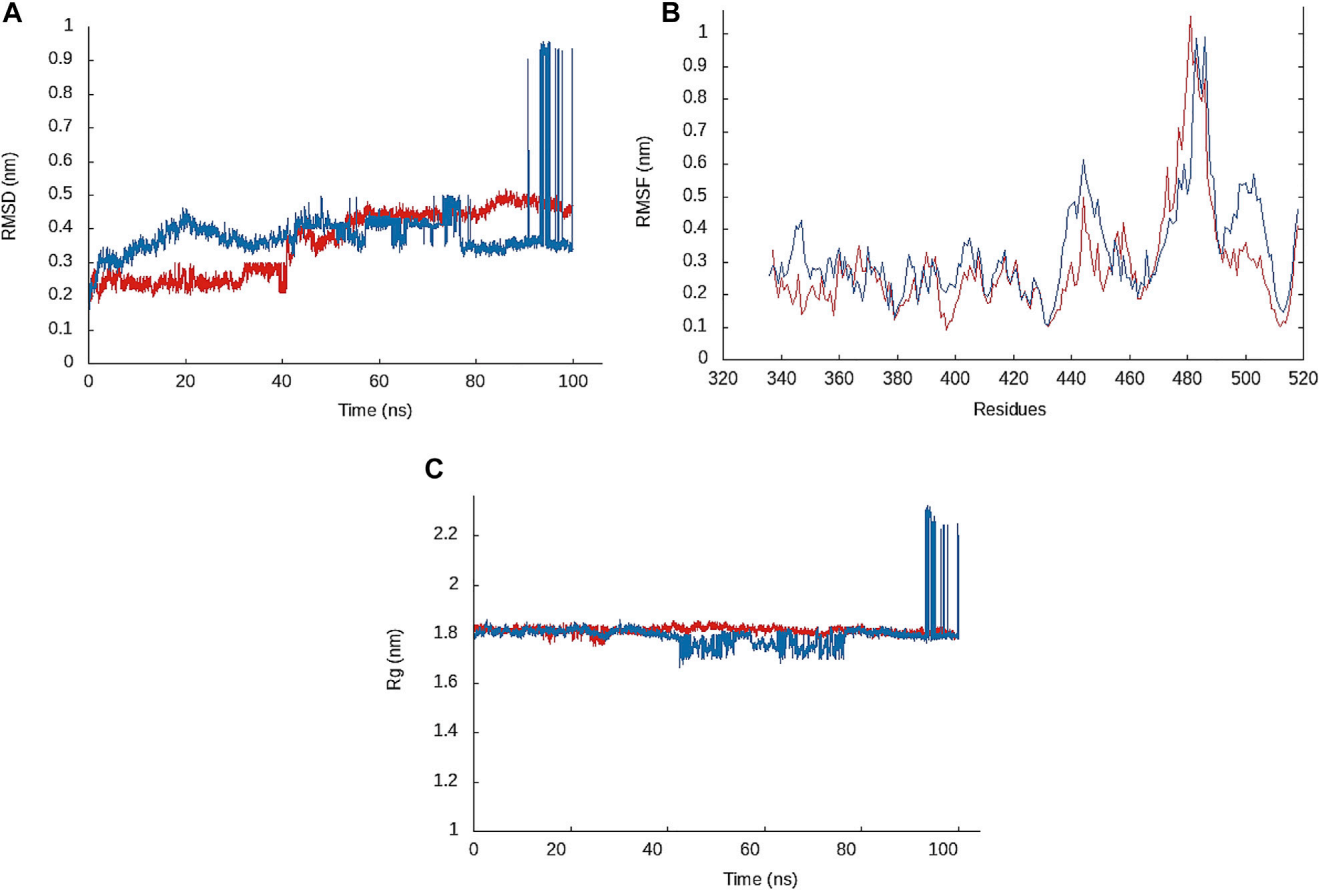
**(A)** RMSD plot of the Pep1-RBD complex (red) and Pep2-RBD complex (blue) backbone atoms. **(B)** Root mean square fluctuation (RMSF) plot showing fluctuation of residues side chains of RBD in complex with Pep1 (red) and Pep2 (blue). **(C)** Radius of gyration (Rg) plot of Pep1-RBD (red) and Pep2-RBD complex (blue).

To investigate the structural stability and dynamic behavior of the designed peptides in complex with RBD, we performed MD simulation of the RBD in complex with Pep1 and Pep2. The docking conformation with lowest energy was subjected to MD simulation. To investigate structural stability of the complex, RMSD plot of the complex backbone was produced. The RMSD values of Pep1-RBD complex remained 0.2–0.25 nm initially for 40 ns and then increased up to 0.4–0.5 nm for 60–100 ns of MD run. Similarly, the RMSD values of Pep2-RBD complex remained 0.3–0.4 nm during initial 90 ns while slightly increased up to 0.95 nm during 90–100 ns (Figure 3A). In general, the RMSD ≤0.3 nm during a 20 ns MD run indicates strong complex stability (Rao et al., 2007; Rani et al., 2016). Overall, a uniform lower RMSD of Pep1-RBD complex indicates that Pep1 bind more tightly to RBD than the Pep2. The RMSD value of Pep1-RBD complex is also lower than the previously reported therapeutic peptide (peptide inhibitor 4: 0.8 nm) for SARS-CoV-2 treatment (Han and Kral, 2020). RMSF determined in the docked complexes shows residues flexibility. The high RMSF values indicate the mobility of residue side chains in relation to their average position (Muralidharan et al., 2021). The RMSF plot of Pep1-RBD complex shows that the residues of RBD at position 358, 417, and 490–500 showing lower fluctuation (nm) than the Pep2-RBD complex. The overall RMSF value of Pep1-RBD complex is less than 0.2 nm in region 1 & II window, which is lower than the RMSF value (0.35 nm) of RBD when bound to intact ACE2 (Basit et al., 2021). The residues involved in binding interaction with lower RMSF values indicates the most stable region of the complex (Ardalan et al., 2018). The lower RMSF values of RBD binding residues indicate that Pep1 form a stable complex with RBD, as RMSF value < 0.4 nm reveals complex stability (Maqsood et al., 2020).

Rg value was determined to describe the structural integrity and folding behavior of the designed peptides in complex with RBD. A low Rg value reveals better structural integrity and folding behavior (Bhowmick et al., 2020; Chatterjee et al., 2020). Pep1-RBD complex showed a uniform and stable Rg value between 1.80–1.84 nm throughout a 100 ns MD run, while the Rg value of Pep2-RBD complex increased to 2.23 nm during 90–100 ns. The overall Rg values for both peptides remained between 1.80–1.84 nm during 0–89 ns, which is lower than the Rg value (2.2 nm) showed by intact ACE2-RBD complex (Basit et al., 2021), which indicates structural integrity of Pep1-and Pep2-RBD complex (Figure 3C). Overall, the MD simulation results suggests that the *de novo* designed peptides form a stabilized complex with RBD and propose their potential to block the SARS-CoV-2 spike glycoprotein for interaction with human cell surface receptor.

## Conclusion

SARS-CoV-2 infects human cells through their receptor binding domain of its spike glycoprotein by interacting with cell surface receptor, ACE2. The *de novo* peptide design opens a new path for producing more potential therapeutic peptides that can mask the RBD critical residues required for interaction with human cell surface receptor, making the SARS-CoV-2 unable to infect human cells. Our *de novo* designed peptides covering 11binding residues of RBD with increased binding affinity and complex stability. A stabilized interactions network was shown by Pep1and Pep2. The designed peptides can be tested experimentally for their binding affinity towards spike glycoprotein, followed by analyzing their potential to inhibit the targeted human cell line from SARS-CoV-2 pseudoparticles infection, live virus infection inhibition in cell culture, followed by assessment of its potential inhibitory activity in animal model of infection.

## Data Availability

The original contributions presented in the study are included in the article/Supplementary Material, further inquiries can be directed to the corresponding authors.
