# Supplementary material for: Designing Short Peptides to Block the Interaction of SARS-CoV-2 and Human ACE2 for COVID-19 Therapeutics
Source: Front Pharmacol. 2021 Aug 27;12:731828. doi: 10.3389/fphar.2021.731828 (PMC8430035; doi:10.3389/fphar.2021.731828)
Supplement: Supplementary file 1 [file DataSheet1.docx]

Supplementary Material


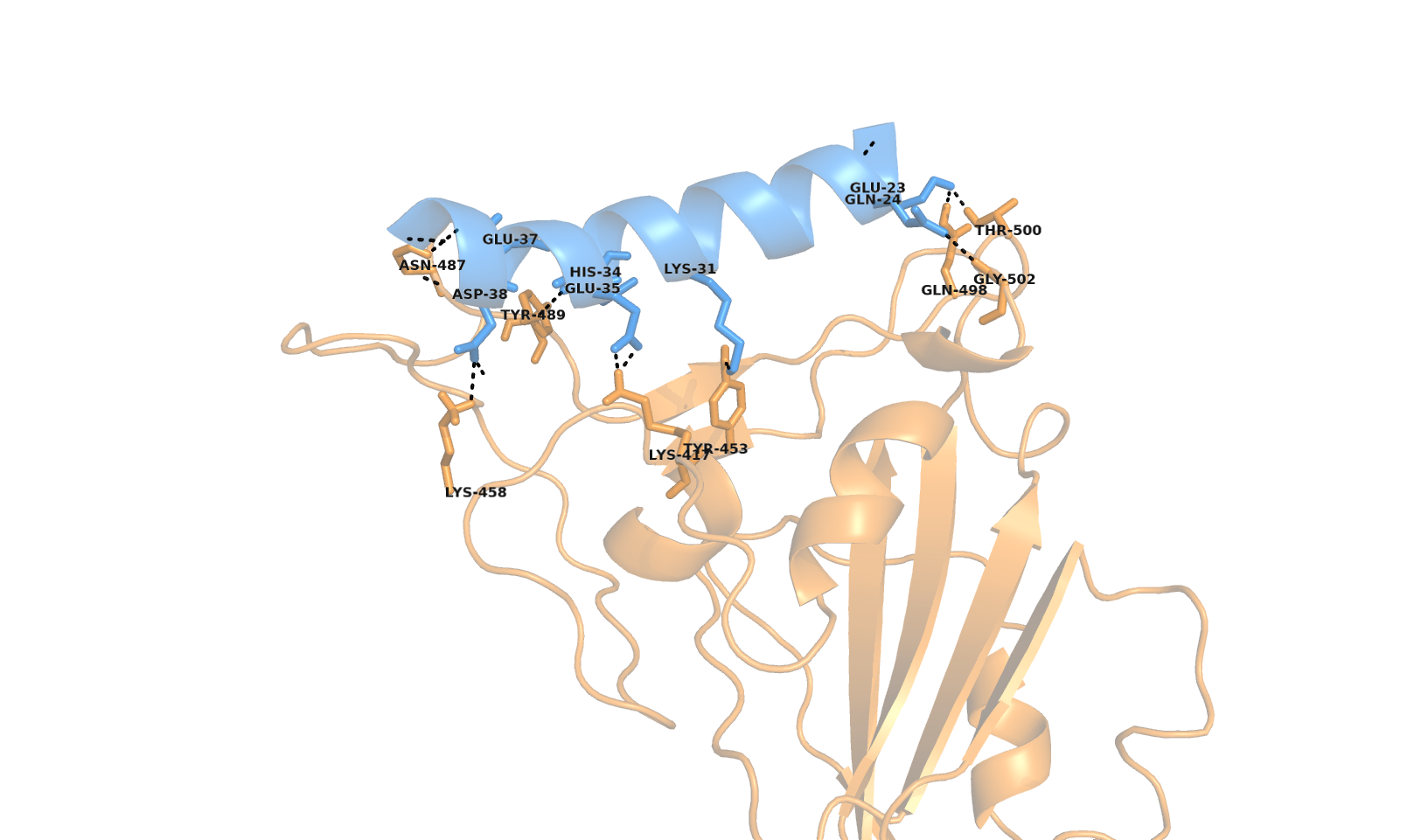

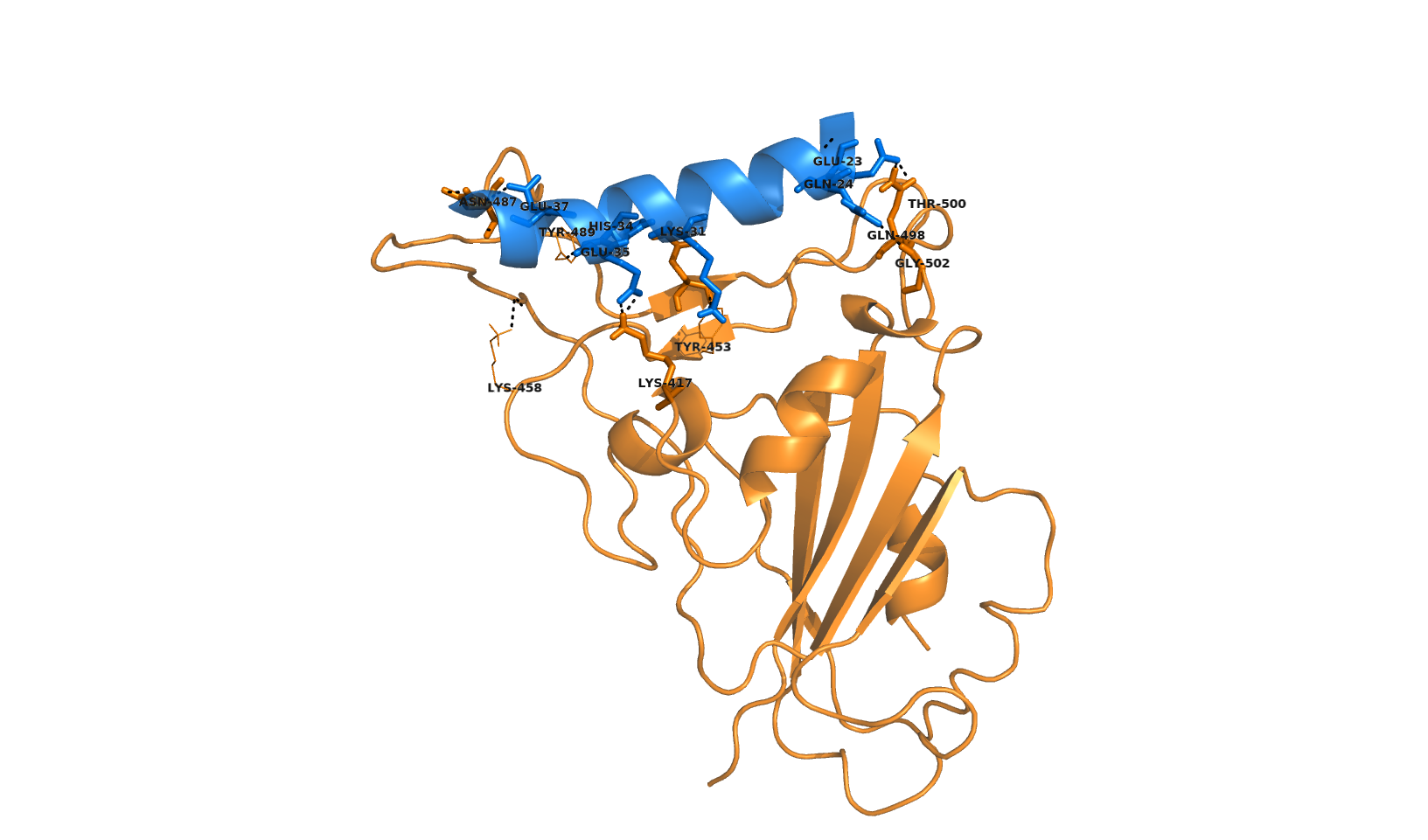


**Supplementary Figure S1.** Docking of tACE2 scaffold region (blue) to RBD (orange) of SARS-CoV-2. The amino acid residues Glu23, Gln24, Lys31, His34, Glu35, Glu37 and Asp38 are showing binding interactions with Tyr453, Lys458, Asn487, Tyr489, Gln498, Thr500 and Gly502 of RBD.


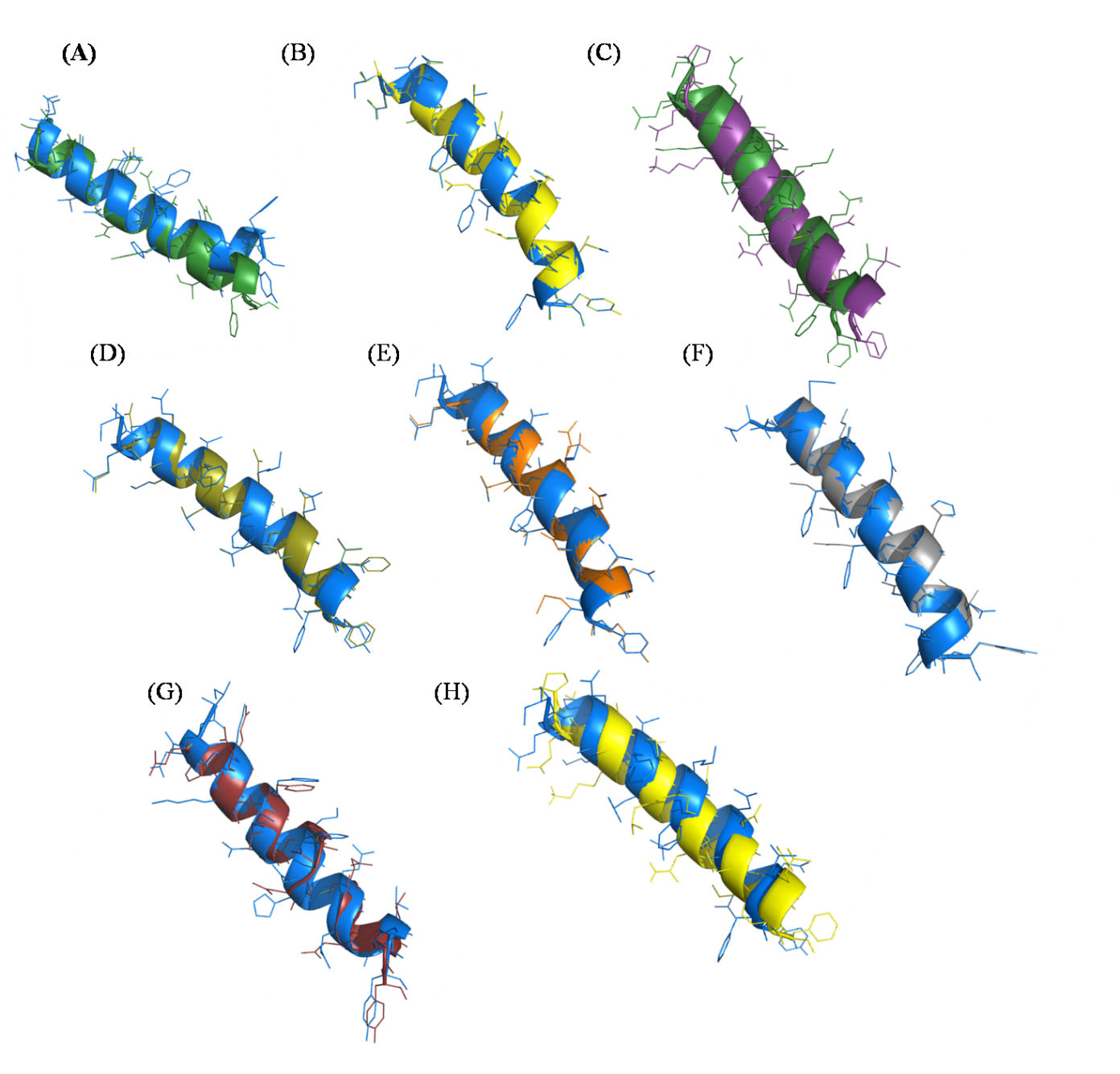


**Supplementary Figure S2.** Superposition of *de novo* designed peptides (Pep3-Pep10) with tACE2 colored as blue. **(A)** Pep3, **(B)** Pep4, **(C)** Pep5, **(D)** Pep6, **(E)** Pep7, **(F)**, Pep8, **(G)** Pep9, and **(H)** Pep10.
